# Impact of SARS-CoV-2 Pandemic and Strategies for Resumption of Activities During the Second Wave of the Pandemic: A Report From Eight Paediatric Hospitals From the ECHO Network

**DOI:** 10.3389/fpubh.2021.630168

**Published:** 2021-04-26

**Authors:** Giuseppe Indolfi, Micol Stivala, Matteo Lenge, Ruben Diaz Naderi, Jennifer McIntosh, Ricard Casadevall Llandrich, Joe Gannon, Kathleen S. McGreevy, Sandra Trapani, Päivi Miettinen, Pekka Lahdenne, Louisa Desborough, Jana Pavare, Annemarie van Rossum, Dagmara Zyska, Massimo Resti, Alberto Zanobini

**Affiliations:** ^1^Meyer Children's Hospital, Firenze, Italy; ^2^Department Neurofarba, University of Florence, Firenze, Italy; ^3^Sant Joan de Deu-Barcelona Children's Hospital, Esplugues de Llobregat, Spain; ^4^European Children's Hospitals Organisation (ECHO), Esplugues de Llobregat, Spain; ^5^Children's Health Ireland, Dublin, Ireland; ^6^HUS New Children's Hospital, Helsinki, Finland; ^7^Great Ormond Street Hospital for Children, London, United Kingdom; ^8^Children's Clinical University Hospital, Riga, Latvia; ^9^Erasmus MC-Sophia Children's Hospital, Rotterdam, Netherlands; ^10^The Children's Memorial Health Institute, Warsaw, Poland

**Keywords:** SARS-CoV-2, preparedness, restart, COVID-19, children

## Abstract

The Severe Acute Respiratory Syndrome CoronaVirus type 2 (SARS-CoV-2) pandemic impacted the organization of paediatric hospitals. This study aimed to evaluate the preparedness for the pandemic among a European network of children's hospitals and to explore the strategies to restart health care services. A cross-sectional, web-based survey was distributed in May 2020 to the 13 children's tertiary care hospitals belonging to the European Children's Hospitals Organisation. Responses were obtained from eight hospitals (62%). Significant reductions were observed in accesses to the emergency departments (41.7%), outpatient visits (35.7%), intensive and non-intensive care unit inpatient admissions (16.4 and 13%, respectively) between February 1 and April 30, 2020 as compared with the same period of 2019. Overall, 93 children with SARS CoV-2 infection were admitted to inpatient wards. All the hospitals created SARS-CoV-2 preparedness plans for the diagnosis and management of infected patients. Routine activities were re-scheduled. Four hospitals shared their own staff with adult units, two designated bed spaces for adults and only one admitted adults to inpatient wards. The three main components for the resumption of clinical activities were testing, source control, and reorganization of spaces and flows. Telemedicine and telehealth services were used before the SARS-CoV-2 pandemic by three hospitals and by all the hospitals during it.

**Conclusion:** The present study provides a perspective on preparedness to SARS-CoV-2 pandemic among eight large European children's hospitals, on the impact of the pandemic on the hospital activities and on the strategies adopted to restart clinical activities.

## Introduction

In December 2019 a novel beta-coronavirus, now officially known as Severe Acute Respiratory Syndrome CoronaVirus type 2 (SARS-CoV-2), emerged in Wuhan, Hubei Province, China, causing a series of severe cases of pneumonia. The virus spread rapidly worldwide and in March 2020 the World Health Organisation (WHO) declared SARS-CoV-2 infection to be a pandemic. As of February 2021, the WHO dashboard (1) reports that there have been more than 111 million confirmed cases and almost 2.5 million deaths; of these, 37 million confirmed cases, including 884 thousand deaths, were reported in European countries (1).

Paediatric cases of SARS-CoV-2 infection accounted for 2% of the total cases diagnosed in China, 1.2% in Italy, and 5% in the United States (2). In children, SARS-CoV-2 infection and the correlated syndrome denominated COronaVIrus DIsease-2019 (COVID-19) is clinically much different from that in adults. According to the clinical categories described by Dong (3), the majority of infected children were asymptomatic or had mild disease and rarely developed severe or critical conditions (4). Children generally had a faster recovery and a better prognosis than adults (5), and deaths were extremely rare. Relatively few pediatric COVID-19 cases were hospitalized and even fewer warranted admission to the paediatric intensive care unit (6–8). Increased hospitalizations were observed in children with underlying long-term medical conditions (LTC), that have higher risk of more severe illness (6–8). Due to the subtle clinical presentation, children were probably less tested and not accurately counted among observed SARS-CoV-2 infection cases. Therefore, although COVID-19 has been described in children at a lower case rate relative to adults, it is likely that the infection rate may be similar (9). Specific to paediatric age, a new inflammatory illness, subsequently named multi-inflammatory syndrome in children and neurological manifestations (including stroke) have been described in children with COVID-19 (10, 11).

The SARS-CoV-2 pandemic has been defined as a mass casualty incident because of its catastrophic effect on public health. The effectiveness of the response to an emergency depends on the availability of adequate human, structural, and economic resources but also on the ability to manage logistics (10). Kandel et al. analysed global operational readiness to prevent, detect and respond to an outbreak of a novel infectious disease, including COVID-19 (10). The 182 countries involved vary widely in terms of preparedness; the authors found that only 57% have strong national and subnational capacities in place (10).

The aims of the present study were to investigate the preparedness for the COVID-19 pandemic among a European network of children's hospitals, determine common strengths, and critically assess any shortcomings. The possible strategies to restart paediatric health care services were analysed, collecting the lessons learned in order to improve and address future preparedness plans.

## Materials and Methods

### The European Children's Hospitals Organisation Network and Survey Participants

The European Children's Hospitals Organisation (ECHO) is a new organisation, established in 2017, representing many of the leading children's hospitals across Europe (https://www.echohospitals.org/). These hospitals take care of some of the most complex patients in Europe and around the world. ECHO's mission is to advocate for children's health and their access to the best quality care through the collaborative work of children's hospitals. This includes joint initiatives to improve the quality of care provided at member hospitals. For this study, a structured survey was conducted within the ECHO network. Thirteen hospitals in 13 different countries (Denmark, France, Germany, Ireland, Island, Israel, Italy, Latvia, Netherland, Norway, Poland, Spain, and United Kingdoms) involved in the management of the COVID-19 pandemic received an emailed invitation to complete the web-based survey [implemented using REDCap (12)] in May 2020. Respondents did not receive any honorarium for completing the survey. One person for each centre completed the survey, the chief executive officer of the hospitals or a delegate of his choice. Ethical approval was not required for this service evaluation study because individual patient data was not collected.

### Survey Content and Development

The survey was written in English and consisted of 85 items divided into 6 sections: (1) general information; (2) preparedness; (3) healthcare professionals; (4) healthcare settings; (5) access to the hospital; (6) preparedness for reopening (see Supplementary Material). The items and the sections were based on the guidelines and recommendations provided by the WHO (13), Centers for Disease Control and Prevention (14) and European Centre for Disease Prevention and Control (15). The survey was piloted with two Italian clinicians/respondents to assess clarity and validity. Only one of the two was among the respondents to the survey. Modifications were made based on feedback and comments. The impact of COVID-19 on hospital outcomes was evaluated by comparing the activity performed between February 1 and April 30 of 2019 (before COVID-19) and 2020 (during COVID-19). The activity was assessed by taking into account outpatient visits, emergency department attendance, non-intensive care unit and intensive care unit inpatient numbers in the two periods.

### Statistical Analysis

Standard descriptive statistics were used to describe response frequency. Missing data were not replaced for this descriptive analysis. Denominators were specified across the result section in order to make clear the response rate. All analyses were conducted using MedCalc Statistical Software version 17.6 (MedCalc Software bvba, Ostend, Belgium; http://www.medcalc.org; 2017).

## Results

Responses were obtained from eight (62%) of the 13 hospitals invited to complete the survey from the following countries: Ireland, Dublin, Children's Health Ireland (Dublin CHI); Finland, Helsinki, HUS New Children's Hospital (Helsinki HUS); Italy, Florence, Meyer Children's Hospital (Florence MCH); The Netherlands, Rotterdam, Erasmus MC-Sophia Children's Hospital (Rotterdam EMC-SCH); Latvia, Riga, Children's Clinical University Hospital (Riga CCUH); Poland, Warsaw, The Children's Memorial Health Institute (Warsaw CMHI); Spain, Barcelona, Sant Joan de Déu Barcelona Children's Hospital (Barcelona SJD); and United Kingdom, London, Great Ormond Street Hospital (London GOSH).

### Characteristics of the Participating Hospitals

The characteristics of the participating hospitals are reported in Supplementary Table A. The median number of inpatient beds per hospital was 314.5 (range 163–596). Seven out of eight hospitals had an emergency department with >8,000 annual attendances (median 43,111, range 8,000–122,382).

### Comparison of the Activities of the Hospitals in 2019 and 2020

Significant reductions were observed when the number of outpatient visits, emergency department attendances, ICU and non-ICU inpatient admissions between February 1 and April 30, 2020 were compared with the same period of 2019 (Table 1). The most relevant reduction rate was observed in accesses to the emergency departments (41.7%) followed by outpatient visits (35.7%), intensive care unit and non-intensive care unit inpatient admissions (16.4 and 13%, respectively).

**Table 1:** Comparison of the activities of the hospitals from February 1 to April 30, 2019 and 2020.

		**Barcelona SJD**	**Florence MCH**	**Helsinki HUS**	**London GOSH**	**Rotterdam EMC-SCH**	**Warsaw CMHI**	**Median**	**Range**
Outpatient visits	2019	64156	74182	13555	60708	56662	58625	59666.5	13555–74182
	2020	37401	50885	13546	39398	53896	29237	38399.5	13546–53896
	Difference (%)	−41.7	−31.4	−0.1	−35.1	−4.9	−50.1	−35.6	-
Emergency department attendances	2019	32123	12366	11687	n.a.	1895	n.a.	6791.0	0–32123
	2020	23257	6129	8758	n.a.	1784	n.a.	3956.5	0–23257
	Difference (%)	−27.6	−50.4	−25.1	-	−5.9	-	−41.7	-
Non-intensive care unit inpatients	2019	5192	2315	2358	10314	4174	9844	4683.0	2315–10314
	2020	4733	1493	1907	7847	3414	6327	4073.5	1493–7847
	Difference (%)	−8.8	−35.5	−19.1	−23.9	−18.2	−35.7	−13.0	-
Intensive care unit inpatients	2019	324	24	512	394	394	345	369.5	24–512
	2020	293	27	400	364	325	270	309.0	27–400
	Difference (%)	−9.6	12.5	−21.9	−7.6	−17.5	−21.7	−16.4	-
Cumulative cases per country^*^	no/100, 000	456.6	335.4	88.8	250.7	219.9	33.3		

*Barcelona SJD, Barcelona, Sant Joan de Déu Barcelona Children's Hospital; Dublin CHI, Dublin, Children's Health Ireland; Florence MCH, Florence, Meyer Children's Hospital; Helsinki HUS, Helsinki, HUS New Children's Hospital; London GOSH, London, Great Ormond Street Hospital; Rotterdam EMC-SCH, Rotterdam, Erasmus MC-Sophia Children's Hospital; Warsaw CMHI, Warsaw, The Children's Memorial Health Institute; n.a., not applicable because these hospitals do not have emergency departments;*

* *Data are from March 1 to April 30, 2020 – source World Health Organization*.

### SARS-CoV-2 Infection Cases

During the study period, 41 confirmed SARS-CoV-2 infection cases were evaluated at the ED, 93 were admitted to paediatric wards, 21 to intensive care units and 13 were transferred to the intensive care unit from another ward within the hospital due to worsening clinical condition. A total of 29 children with SARS-CoV-2 infection were admitted to the hospital for different reasons and found to be infected during hospitalization. Data according to each hospital are summarized in Table 2.

**Table 2:** SARS-CoV-2 infection cases in the 8 hospitals from February 1 to April 30, 2020.

	**Barcelona SJD**	**Florence MCH**	**Helsinki HUS**	**London GOSH**	**Riga CCUH**	**Rotterdam EMC-SCH**	**Warsaw CMHI**
Evaluated at emergency departments	28	3	3	0	7	0	0
Admitted to wards	41	13	0	33	6	0	0
Admitted to intensive care units	3	0	0	18	0	0	0
Transferred to intensive care units^*^	1	0	0	11	0	1	0
Admitted for other reasons	10	1	0	17	0	1	0

*Barcelona SJD, Barcelona, Sant Joan de Déu Barcelona Children's Hospital; Florence MCH, Florence, Meyer Children's Hospital; Helsinki HUS, Helsinki, HUS New Children's Hospital; London GOSH, London, Great Ormond Street Hospital; Riga CCUH, Riga, Children's Clinical University Hospital; Rotterdam EMC-SCH, Rotterdam, Erasmus MC-Sophia Children's Hospital; Warsaw CMHI, Warsaw, The Children's Memorial Health Institute; m.d., missing data; n.a., not applicable;*

* *Patients transferred from another ward for worsening clinical condition*.

### Preparedness Plan-Hospital Strategies

All the eight involved hospitals created a multidisciplinary planning committee that developed written SARS-CoV-2 infection preparedness plans for the evaluation, diagnosis, and management of confirmed or suspected COVID-19 patients. SARS-CoV-2 infection communication plans were also developed by all hospitals in order to share information and relevant policies with department/unit heads, facility staff, families, volunteers, and other people accessing the facility. Routine activities, such as elective procedures, non-urgent outpatient visits, ancillary exams, or training or scientific sessions for health professionals were re-scheduled or cancelled. Two hospitals (25%) designated bed spaces for adults with COVID-19 and one (12.5%) admitted adults with COVID-19 in both intensive care unit or non- intensive care unit beds. A separate emergency department area for the triage, assessment and management of children with fever or respiratory symptoms was created by five out of six hospitals (83.3%). All the hospitals identified a designated area to admit and isolate patients with suspected COVID-19 and a specific ward to admit and isolate patients with known SARS-CoV-2 infection. The hospital that admitted adults with COVID-19, also increased the number of airborne infection isolation rooms by 10 units. Less than 50% of these rooms were used at the same time. Two hospitals increased the number of intensive care unit beds with mechanical ventilators (one by 25%, the other by 48%), which were not actively used in the reporting period. All hospitals limited the number of parents or caregivers to one person per patient; three (37.5%) created a specific register of all visitors who entered and exited the room of a subject with confirmed/suspected SARS-CoV-2 infection. All parents or caregivers were screened for fever or respiratory symptoms in three (37.5%) and five (62.5%) hospitals, respectively. Nasopharyngeal swabs were performed on all parents in one hospital (1/7; 14.3%) and only in selected cases in the remaining six (6/7; 85.7%).

### Care of Patients With LTC or Special Healthcare Needs (SHCN)

In order to continue the routine care for non-COVID-19 patients with LTC or SHCN, different strategies were adopted. All the hospitals (n 7) identified and maintained the essential services that the hospital provides at all times and under any circumstances. Five hospitals (5/7; 71.4%) developed resources for children living with LTC or SHCN, such as print- and web-based educational materials and access to support telephone lines. Six hospitals (6/7; 85.7%) proactively reviewed LTC or SHCN patients requiring care and their possible needs if healthcare services were disrupted or identified clear points of contact for LTC and SHCN. Three hospitals (3/7; 42.9%) reached out to family paediatricians or organizations/foundations to provide local specific advice or services. Four hospitals (4/7; 57.1%) provided mental health resources to help parents manage stress, employ coping strategies and promote adaptive behaviour changes.

### Health Care Professionals

Strategies for testing SARS-CoV-2 infection in health care professionals are summarized in Table 3. Adopted strategies for screening were nasopharyngeal swab (6/7; 85.7%), serologic tests (2/7, 28.6%), or both (1/7, 14.3%). Healthcare professionals were also supported by mental health resources to help manage stress, employ coping strategies and promote adaptive behaviour change (5/7; 71.4%) and were trained (and retrained) on personal protective equipment to acquire competency with selection and proper use (7/7; 100%). In four hospitals (57.1%), the paediatric staff helped the staff of adult units to cover shortages, including providing adult care within their own hospitals or providing care at an outside adult hospital.

**Table 3:** Strategies for testing SARS-CoV-2 infection on health care professionals.

**Health care professionals**	*n* (%)^*^
Testing only workers who developed signs or symptoms compatible with COVID-19	3 (42.9%)
Testing selected workers belonging to specific exposure risk categories	1 (14.3%)
Testing all workers at the hospital (including non-clinical staff)	1 (14.3%)

* *Data available from seven paediatric hospitals*.

### Strategies for Resumption of Clinical Activities

The three main components of the strategic planning process for the resumption of clinical activities were testing, source control, and reorganization of spaces and flows of patients and are summarized in Supplementary Table B. Four out of seven hospitals (57.1%) will permanently maintain a designated ward/unit for patients with confirmed SARS-CoV-2 infection. Centralization of children with known COVID-19 to a single local/regional referral hospital was considered reasonable and effective by three hospitals (3/7; 42.8%); four (57.1%) created a trained in-hospital contact tracing program and workforce. All the hospitals have developed a stepwise approach to restart all healthcare services as described in Table 2.

### Telemedicine and Telehealth

Telemedicine and telehealth services were used before the SARS-CoV-2 pandemic by three (3/8; 37.5%) hospitals and by all the hospitals during it.

## Discussion

The present study, based on a survey completed by eight European children's hospitals, explored the preparedness for COVID-19, the impact of the pandemic on hospital activities, and the strategies adopted to restart the paediatric health care services. The main pillars of preparedness response were homogeneous across the different hospitals. Overall, a significant reduction in the number of inpatients, outpatients, and attendances to the emergency departments was observed. Different approaches were used for reopening and the resumption of activities.

Preparedness for health emergencies includes all plans developed in anticipation of a crisis and is essential to provide an effective response to pandemics. In all of the children's hospitals involved in the survey, a preparedness plan was developed and implemented to ensure a rapid and effective response to COVID-19, to provide care to a possible high volume of SARS-CoV-2 infected children and to prevent nosocomial outbreaks. Most of the measures applied were inspired by the adult models and in line with the guidelines released by the major international organizations (13–15). The response was similar across the different hospitals. In all the hospitals, a written SARS-CoV-2 infection preparedness plan for the evaluation, diagnosis, and management of confirmed or suspected COVID-19 was put in place and communicated to staff, patients, and families with the main aims of protecting patients, healthcare personnel and visitors from COVID-19. Routine non-urgent activities were postponed and only essential services were maintained. Access to the facility was restricted to one parent/caregiver per patient, assessed for fever or respiratory symptoms. All the hospitals reorganized spaces and flows of patients. Children with suspected or known SARS-CoV-2 infection were admitted to dedicated areas of the emergency department and moved to dedicated inpatient wards. Healthcare professionals were trained and retrained in the correct use of personal protective equipment and were tested and retested for SARS-CoV-2 with nasopharyngeal swabs, serological tests or both.

It was of interest to evaluate whether the response plan was oriented toward optimizing services and adopting alternative models of care as per WHO recommendations (13). Telemedicine, the practice that allows healthcare professionals to evaluate, diagnose, and treat patients at a distance using telecommunications technology, was implemented as a possible solution to overcome the barriers of social distancing. Only three hospitals were providing telemedicine prior to the pandemic reflecting the low investments in digital health in the past. Health information and digital health solutions are playing a key role in coping with the ongoing COVID-19 outbreak contributing positively to the resilience of the health service delivery system. Future investments should certainly take communicable diseases into consideration and telemedicine as an essential tool to ensure the provision and continuity of care reducing face-to-face contact (16, 17).

In the pandemic scenario, children with disabilities or chronic conditions, including respiratory diseases, immunodeficiencies and cancers, are highly vulnerable (18). These children require continuous monitoring, assistance and access to treatments but, at the same time, need to be protected from possible exposure to the virus as they are at increased risk of severe illness (16, 18). The preparedness response for children with LTC or SHCN among the eight children's hospitals was variable. Improvements could come from informing individuals as to what their level of risk may be so they can make individual decisions about illness prevention and from increasing the awareness of hospitals and clinicians in order to develop specific resources.

COVID-19 has been recognized as a traumatic experience directly influencing the mental health of children. Social isolation and loneliness were associated with anxiety and depression in children and adolescents. Mental health support to help patients and families to manage COVID-19 stressors was provided by only four hospitals involved in the survey. Indeed, even in view of a possible second wave of the pandemic, it is necessary to strengthen the in-hospital mental health resources, implementing mechanisms for surveillance and intervention and expanding telehealth-based modalities (19).

Children and adolescents are overall less severely affected by COVID-19 than adults and elderly patients. The evaluation of the clinical outcome of SARS-CoV-2 infection in children and adolescents was beyond the scope of this article. Overall, in the 3-month study period, the total number of children evaluated and admitted with COVID-19 was low and even fewer required admission to the ICU (20). The clinical impact of infection in children and the burden of the disease on the clinical activities of children's hospitals was unknown at the beginning of the pandemic. Preparedness plans were oriented toward providing care to a possible high volume of SARS-CoV-2 infected children. Only two hospitals have designated bed spaces for adults with COVID-19 and only one admitted adults with COVID-19. The paediatric staff of four hospitals helped the staff of adult units to cover shortages. The present data, as previously reported, confirm that the COVID-19 emergency proved to be more logistical than clinical in children, requiring more organizational efforts than medical assistance (21, 22). At the same time, the eight hospitals that completed the survey experienced substantial decreases in all admissions, in comparison to the same period in 2019, especially to emergency departments, consistent with previous reports. Lazzerini et al. found a reduction ranging from 73 to 88% in Italian paediatric emergency department visits in the period March 1–27, 2020, compared to 2019 and 2018 (23). Ciofi Degli Atti (24) reported a 68% reduction in the mean daily emergency department visits and a 31% reduction in urgent hospitalizations from January 1 to April 20, 2020 compared to the 2 months before (24). A significant reduction in the number of outpatient visits, among the other routine activities of the hospitals involved in this study, was observed. The decrease in the number of outpatient visits was less evident where the burden of the pandemic during the study period was lower such as in Helsinki and Rotterdam. It could not be excluded that differences in the hospital organization and in the country approach to the pandemic could have influenced these results. Concerns have been raised that the number of children who delay seeking medical attention, from vaccines to surgery, is increasing (25). This delayed and late presentation has been defined as “collateral damage” due to an unintended effect of lockdown and social distancing that is more harmful for children's health than the virus itself (26). Sporadic cases of delayed access to care for children with severe diseases (25, 27, 28) and adults with fatal conditions such as strokes (29) or cardiac diseases (30) have been reported. In both adults and children, reluctance to seek medical care is probably correlated with restriction measures and with the fear of potential exposure to COVID-19 (23).

After the critical phase of strict social distancing and lockdown, the strategies for the resumption of clinical activities were investigated. Although all the participating hospitals have planned a stepwise approach for the resumption of activities in the safest possible way, different strategies were applied. While there was an agreement with regard to the reorganization of spaces and flows of patients with active source control measures, major differences were observed in the testing approach to patients, caregivers and healthcare professionals. Nasopharyngeal swabs, the preferred testing strategy for healthcare professionals, were performed on all parents/caregivers entering the facility only in one hospital. It can be speculated that the different timing and evolution of the pandemic across European countries may have impacted the strategic approach of each hospital, influencing the response at the time of the survey.

In view of the low number of children admitted in the first phase of the pandemic and of the restrictions to routine hospital activities, centralization of children with known SARS-CoV-2 infection to a single referral hospital was explored as a possible strategy. Although this approach has been successfully implemented and described before (24, 31), only three hospitals expressed a favourable opinion. Whether centralizing children with known SARS-CoV-2 infection is effective in order to optimize workforce and resources, decrease the risk of delayed access to care and reduce the disruption of services, especially for children with underlying chronic conditions, should be evaluated.

The present study has some limitations. First, it reports on the approach to the pandemic of a limited number of hospitals from different countries with different SARS-CoV-2 epidemiology. Furthermore, a hospital's overall service delivery environment is heavily influenced by its basic infrastructure, a factor that could have impacted the responses. Finally, the response rate to the survey was high but, as expected, not complete. Despite these limitations, our study provides for the first time a perspective on COVID-19 preparedness among eight large European children's hospitals, on the impact of the pandemic on the hospital activities and, more importantly, on the strategies adopted to restart the paediatric health care services.

## Data Availability Statement

The original contributions presented in the study are included in the article/Supplementary Material, further inquiries can be directed to the corresponding authors.

## Author Contributions

GI, MS, ML, and ST: conceptualization. GI, MS, ML, ST, RDN, JM, and KSM: methodology. RL, JG, ST, PM, PL, LD, JP, AR, and DZ: acquisition of data. GI, MS, and ML: data curation, formal analysis, and writing-original draft. GI, RDN, MR, and AZ: supervision. GI, JG, PM, PL, LD, JP, AR, DZ, MR, and AZ: writing-review and editing. All authors contributed to the article and approved the submitted version.

## Conflict of Interest

The authors declare that the research was conducted in the absence of any commercial or financial relationships that could be construed as a potential conflict of interest.
