# Supplementary material for: Impact of SARS-CoV-2 Pandemic and Strategies for Resumption of Activities During the Second Wave of the Pandemic: A Report From Eight Paediatric Hospitals From the ECHO Network
Source: Front Public Health. 2021 Apr 26;9:630168. doi: 10.3389/fpubh.2021.630168 (PMC8107357; doi:10.3389/fpubh.2021.630168)
Supplement: Supplementary file 1 [file Data_Sheet_1.PDF]

Article title: Impact of SARS-CoV-2 Pandemic and Strategies for Resumption of Activities During the Second Wave of the Pandemic: a Report from Eight Paediatric Hospitals from the ECHO Network

Supplementary material

Page 2: table A
Page 3-4: table B
Page 5-18: survey

Table A. Characteristics of the participating hospitals

	Barcelona SJD	Dublin CHI	Florence MCH	Helsinki HUS	London GOSH	Riga CCUH	Rotterdam EMC-SCH	Warsaw CMHI
Inpatient beds	319	436	250	163	425	310	167	596
Emergency Department	yes	yes	yes	yes	no	yes	yes	no
Annual attendances	122382	m.d.	43111	40300	n.a.	64993	8000	n.a.
Airborne Infection Isolation Rooms	2	m.d.	11	9	68	5	9	0
Intensive care unit beds with mechanical ventilators	24	m.d.	12	45	48	10	60	24

Note: Barcelona SJD, Barcelona, Sant Joan de Déu Barcelona Children's Hospital; Dublin CHI, Dublin, Children's Health Ireland; Florence MCH, Florence, Meyer Children's Hospital; Helsinki HUS, Helsinki, HUS New Children's Hospital; London GOSH, London, Great Ormond Street Hospital; Riga CCUH, Riga, Children's Clinical University Hospital; Rotterdam EMC-SCH, Rotterdam, Erasmus MC-Sophia Children's Hospital; Warsaw CMHI, Warsaw, The Children's Memorial Health Institute; m.d., missing data; n.a., not applicable

Table B. Strategies for the resumption of clinical activities

**SARS-CoV-2 testing**	**n (%)**
Increasing testing capacity	4 (57)
Testing and retesting healthcare workers	4 (57)
Testing and retesting inpatients with fever and or respiratory symptoms	5 (71)
Testing and retesting all inpatients	2 (29)
Testing and retesting outpatients with fever and or respiratory symptoms	3 (43)
Testing all outpatients	0
Antibody testing for the healthcare workers	1 (14)
Testing all surgical patients	1 (14)
Testing high risk inpatients	1 (14)
**Source Control**	
Screening fever and respiratory symptoms for all visitors at entry in the hospital	2 (29)
Requiring everyone entering the facility to wear a facemask or cloth face covering	4 (57)
Requiring healthcare workers to wear a facemask at all times while they are in the hospital	4 (57)
Instructing outpatients to call ahead and discuss the need to reschedule their appointment for routine medical care if they develop fever or symptoms of SARS-CoV2 infection	5 (71)
Providing instructions to and supplies for respiratory hygiene and cough etiquette, hand hygiene	4 (57)
Strengthening coordination between primary healthcare, social services and hospital	2 (29)
**Reorganization of spaces and flows**	
Limiting and monitoring points of entry to the facility	5 (71)
Creating physical barriers (e.g., glass, plastic windows or curtains) at reception, triage and shared areas to limit close contact between patients and between triage personnel and patients	5 (71)
Implementing air-handling systems	1 (14)
Permanently establishing separate flow for patients with suspected SARS-CoV2 infection	5 (71)

Maintaining a designated ward/unit for patients with suspected SARS-CoV2 infection	5 (71)
Maintaining a designated ward/unit for patients with confirmed SARS-CoV2 infection	4 (57)

Note: data are n (%); data available from seven paediatric hospitals

ECHO COVID-19 assessment

Thank you for your participation in this initiative to gather the best evidence and expert opinions regarding the approach of the ECHO hospitals to the SARS-CoV-2 pandemic.

The objectives of this survey are to:

- Ascertain the impact of SARS-Cov-2 infection and COVID-19 in your hospital. In Section 1 you will be asked to provide data on the activities of your hospital from February 1st and April 30th 2020 and during the same time period in 2019

- Evaluate the preparedness of the ECHO hospitals to the COVID-19 pandemic [Sections 2-5]

- Obtain specific and valuable information on the strategies that your hospital is or will apply for reopening and restarting after the lockdown [Section 6]

Please note that you can leave the survey at any time and come back later to complete your responses. To do this, choose the “Save and return later” option (bottom of the screen) and write down the code you are given. When you re-open the survey at the link above, click on “Returning?” (top right) and enter your code, to re-open your partially completed survey and continue.

If you have any queries or comments about the questionnaire, please contact: matteo.lenge@meyer.it

SECTION 1: GENERAL INFORMATION

1 Date: ________________

2 Name of the CEO: ________________

3 Name of the main researcher involved in the survey: ________________

4 Contact email address of the main researcher: ________________

5 Institution - City
◯ Sant Joan de Déu Barcelona Children's Hospital - Barcelona ◯ Rigshospitalet - Copenhagen ◯ Children's Health Ireland - Dublin ◯ Meyer Children's Hospital - Florence ◯ HUS New Children's Hospital - Helsinki ◯ Great Ormond Street Hospital for Children - London ◯ Dr. von Hauner Children's Hospital, LMU - Munich ◯ Oslo University Hospital Division of Paediatrics - Oslo ◯ Necker-Enfants Malades Universitary Hospital AP-HP - Paris ◯ Schneider Children's Medical Center of Israel - Petach-Tikva ◯ Children's Clinical University Hospital - Riga ◯ Erasmus MC-Sophia Children's Hospital - Rotterdam ◯ The Children's Memorial Health Institute - Warsaw

6 Country: ________________

7 Number of in-patient hospital beds prior to implementing the COVID-19 response plan: ________________

8 Does your hospital have an Accident and Emergency Department? ◯ Yes ◯ No

8.1 If yes, number of annual attendances (2019): ______________________

9 Are Airborne Infection Isolation Rooms (AIIRs) available at your hospital? ◯ Yes ◯ No

9.1 If yes, number of AIIRs (prior to implementing the COVID-19 response plan): ______________________

10 Is an Intensive Care Unit (ICU) available at your hospital? ◯ Yes ◯ No

10.1 If yes, number of ICU beds with available mechanical ventilators (prior to implementing the COVID-19 response plan): ______________________

11 Was your hospital regularly using telemedicine and tele-health before the COVID-19 outbreak?

◯ Yes ◯ No

SECTION 1: GENERAL INFORMATION

12 From February 1st to April 30th, 2019 could you please provide the number of:

12.1 Outpatient visits: ______________________

12.2 ED attendances: ______________________

12.3 Inpatients (non-ICU): ______________________

12.4 Inpatients ICU: ______________________

SECTION 1: GENERAL INFORMATION

13 From February 1st to April 30th, 2020 could you please provide the number of:

13.1 Outpatient visits: ______________________

13.2 ED attendances: ______________________

13.3 Inpatients (non-ICU): ______________________

13.4 Inpatients ICU: ______________________

SECTION 1: GENERAL INFORMATION

14 From February 1st to April 30th, 2020 could you please provide the number of children with confirmed SARS-CoV-2 infection who were:

14.1 Evaluated at the ED: ________________________

14.2 Admitted to paediatric wards: ________________________

14.3 Admitted to ICU: ________________________

14.4 Transferred to the ICU from another ward for worsening clinical conditions: ________________________

15 How many children admitted for a different reason between February 1st to April 30th, 2020 have been diagnosed with SARS-CoV-2 infection? ________________________

SECTION 2: PREPAREDNESS FOR COVID-19

1 Has a multidisciplinary planning committee or team been created to specifically address COVID-19 preparedness plan?

○ Yes ○ No

2 Has your children's hospital developed a written COVID-19 preparedness plan for the evaluation, diagnosis and management of confirmed or suspected COVID-19 patients?

○ Yes ○ No

3 Has a COVID-19 communication plan been developed in order to share information and relevant policies with departments/units heads, facility staff, families, volunteers and other persons coming into the facility?

○ Yes ○ No

4 When did COVID-19 preparedness planning start within your hospital? This refers to planning specific to the COVID-19 pandemic and not to the development of general emergency preparedness response planning. ______________________________

5 Which strategies did your plan include for continuing routine care for non COVID-19 patients with long term conditions (LTCs) or special healthcare needs (SHCN)? Please indicate new activities that were initiated in response to COVID-19. Check all that apply.

☐ - identify and maintain the essential services that the hospital provides at all times and under any circumstances ☐ develop resources for children living with LCTs or SHCN such as print and web-based educational materials and access to support telephone lines ☐ proactive review LCTs or SHCN patients requiring care and their possible needs if healthcare services are disrupted ☐ identify a clear point of contact for LCTs or SHCN patients care ☐ reach out to family pediatricians or to peer support organizations/fundations for providing local specific advices or services ☐ other

5.1 If other, please specify

SECTION 3: HEALTHCARE WORKERS (I.E. DOCTORS, NURSES, LABORATORY, WORKERS, FACILITY OR MAINTANANCE WORKERS, CLINICAL TRAINEES, VOLUNTEERS)

1 Have you defined a strategy to assist with assessment, monitoring and work restriction decisions for health care professionals with potential exposure to COVID-19? ◯ Yes ◯ No

1.1 If yes, which criteria were used to test healthcare workers?

◯ exposure risk category
◯ only if healthcare workers developed signs or symptoms compatible with COVID-19
◯ all healthcare workers were or are being tested
◯ other

1.1.1 If other, please specify

1.2 If yes, your screening strategy included (check below categories):

☐ nasopharyngeal swab ☐ serologic tests ☐ nasopharyngeal swab and serologic tests
☐ other

1.2.1 If other, please specify

2 Has your hospital provided mental health resources to help healthcare professionals manage stress, employ coping strategies and promote adaptive behaviour change?

◯ Yes ◯ No

3 Did the paediatric staff of your hospital help the staff of adult units to cover for shortages from February 1st to April 30th, 2020? This may include providing adult care within your hospital or providing care at an outside adult hospital.

◯ Yes ◯ No

4 Have healthcare workers received from February 1st to April 30th, 2020 job-specific training and retraining on personal protective equipment to acquire competency with selection and proper use?

◯ Yes ◯ No

SECTION 4: HEALTHCARE SETTINGS

1 If any, which of the following activities have you re-scheduled or cancelled due to the COVID-19 pandemic (check all that apply):

☐ elective procedures ☐ elective surgeries ☐ out-patient non urgent visits ☐ ancillary exams
☐ training or scientific sessions for health professionals

2 Has your hospital designated bed spaces for adults with COVID-19?

◯ Yes ◯ No

3 Has your hospital admitted adults with COVID-19? ◯ Yes ◯ No

3.1 If yes, check all that apply:

☐ ICU ☐ non-ICU

4 Have you created a separate area in the Accident and Emergency Department for the triage, assessment and management of patients with fever or respiratory symptoms?

◯ Yes ◯ No ◯ Not applicable

5 Have you identified a designated area to admit and isolate patients with suspected COVID-19?

◯ Yes ◯ No

6 Have you identified a designated ward to admit and isolate patients with known COVID-19?

◯ Yes ◯ No

7 Has your hospital increased the total number of Airborne Infection Isolation Rooms (AIIRs) since you implemented your COVID-19 response plan? ◯ Yes ◯ No

7.1 If yes, how many AIRRs has your hospital added? ______________________ (number)

7.2 If the number of AIRRs changed during the reporting period, report the highest number of open AIRRs ______________________ (number)

7.3 If yes, have you used the AIRRs that were added? ◯ Yes ◯ No

7.3.1 If yes, what percentage of your AIRRs were occupied between February 1st to April 30th 2020?

◯ < 50% ◯ > 50%

8 Has your hospital increased the number of ICU beds with available mechanical ventilators since you implemented your COVID-19 response plan? ◯ Yes ◯ No

8.1 If yes, how many ICU beds has your hospital added? ______________________ (number)

8.2 If the number of ICU beds changed during the reporting period, report the highest number of open ICU beds: ______________________ (number)

8.3 If yes, have you used the ICU beds that were added? ◯ Yes ◯ No

8.1.1 If yes, what percentage of your ICU beds were occupied between Feb 1st - April 30th 2020?

◯ < 50 %   ◯ > 50 %

9 Has your hospital maintained a register of all visitors who entered and exited the room of a confirmed or suspected COVID-1 patient for the purpose of contact tracing?

◯ Yes   ◯ No

SECTION 5: MANAGEMENT OF PARENTAL AND CAREGIVERS ACCESS AND MOVEMENT SINCE THE IMPLEMENTATION OF THE COVID-19 RESPONSE PLAN

1 Did your hospital limit the number of parents or caregivers able to be with a patient?

◯ Yes, one parent or caregiver per patient ◯ No ◯ Other

1.1 If other, please specify ________________________________

2 Has your hospital actively screened all parents or caregivers for fever upon entry to the healthcare facility?

◯ Yes ◯ No

3 Has your hospital actively screened all parents or caregivers for respiratory symptoms of COVID-19 upon entry to the healthcare facility?

◯ Yes ◯ No

4 Has your hospital collected on nasopharyngeal swabs to parents or caregivers prior to entering the healthcare facility? ◯ Always ◯ Only in selected cases

4.1 If in selected cases, please specify

5 Has your hospital provided mental health resources to help parents manage stress, employ coping strategies and promote adaptive behavior changes?

◯ Yes ◯ No

6 If you could modify something in your hospital's approach to this pandemic, what would you change?

SECTION 6: PREPAREDNESS TO RESTART AFTER LOCKDOWN

1 Which of the following strategies is your hospital applying to restart activities after lockdown? Check all that apply.

☐ TESTING
☐ SOURCE CONTROL
☐ REORGANIZATION OF SPACES AND FLOWS

1.1 TESTING

☐ increasing SARS-CoV-2 testing capacity ☐ SARS-CoV-2 testing and retesting healthcare workers ☐ SARS-CoV-2 testing and retesting inpatients with fever and or respiratory symptoms ☐ SARS-CoV-2 testing and retesting all inpatients ☐ SARS-CoV-2 testing outpatients with fever and or respiratory symptoms ☐ SARS-CoV-2 testing all outpatients ☐ other

1.1.1 If other, please specify: ______________________

1.2 SOURCE CONTROL

☐ screening fever and respiratory symptoms for all visitors at entry in the hospital ☐ requiring everyone entering the facility to wear a facemask or cloth face covering ☐ requiring healthcare workers to wear a facemask at all times while they are in the hospital ☐ instructing outpatients to call ahead and discuss the need to reschedule their appointment for routine medical care if they develop fever or symptoms of COVID-19 ☐ providing instructions to and supplies for respiratory hygiene and cough etiquette, hand hygiene ☐ strengthening coordination between primary healthcare, social services and hospital ☐ other

1.2.1 If other, please specify: ______________________

1.3 REORGANIZATION OF SPACES AND FLOWS

☐ limiting and monitoring points of entry to the facility ☐ creating physical barriers (e.g., glass, plastic windows or curtains) at reception, triage and shared areas to limit close contact between patients and between triage personnel and patients ☐ implementing air-handling systems ☐ permanently establishing separate flow for patients suspected SARS-CoV2 infection ☐ maintaining a designated ward/unit for patients with suspected SARS-CoV2 infection ☐ other

1.3.1 If other, please specify: ______________________

2 Given your experience with the first phase of COVID-19 epidemic, do you think that centralization of children with known COVID-19 to a single local/regional referral hospital could be reasonable and effective?

◯ Yes ◯ No

2.1 If yes, please, add any comment you would feel important

3 Do you plan on permanently maintaining a designated ward/unit for patients with confirmed COVID-19?

◯ Yes ◯ No

4 Do you plan on maintaining and expanding telemedicine and tele-health services in your hospital?

◯ Yes ◯ No

5 Is your hospital building a trained in-hospital contact tracing programme and workforce?

◯ Yes ◯ No

6 Have you established alternate care sites where children and their caregiver / parent with confirmed COVID-19 can remain and receive medical care for the duration of their isolation period?

◯ Yes ◯ No

7 Has your hospital developed a stepwise approach to responsibly restart all the healthcare services? ◯ Yes ◯ No

7.1 If yes, please could you briefly explain your strategy?

____
